# Features of Clinical Manifestations and Heart Rate Variability in Children with Malignant Vasovagal Syncope

**DOI:** 10.3390/children12050636

**Published:** 2025-05-15

**Authors:** Wenrui Xu, Chunyu Zhang, Junbao Du, Hongfang Jin, Ying Liao

**Affiliations:** 1 Department of Pediatrics, Peking University First Hospital, Beijing 100034, China; xuwenrui@pku.edu.cn (W.X.); chunyudoctor@pku.edu.cn (C.Z.); drjunbaodu@pku.edu.cn (J.D.); jinhongfang@bjmu.edu.cn (H.J.); 2 State Key Laboratory of Vascular Homeostasis and Remodeling, Peking University, Beijing 100191, China

**Keywords:** children, heart rate variability, malignant vasovagal syncope, risk factor

## Abstract

**Background:** This study aimed to identify the risk factors associated with malignant vasovagal syncope (VVS), a rare yet clinically significant subtype of VVS. **Methods:** This single-center case–control study enrolled children diagnosed with malignant VVS, and the malignant VVS patients were matched in a 1:4 ratio with non-asystolic VVS children as a control group through age and sex stratification. Clinical characteristics and heart rate variability (HRV) parameters were analyzed. Binary logistic regression analyses were used to identify the risk factors significantly associated with malignant VVS. **Results:** A total of 10 patients in the malignant group and 40 children in the control group were included. The malignant group exhibited earlier symptom onset (7.0 ± 2.7 vs. 9.7 ± 2.7 years, *p* < 0.05) than the control group, and children in the malignant group had a higher prevalence of central triggers (60.0% vs. 17.5%, *p* < 0.05) and convulsive/incontinence episodes (80.0% vs. 17.5%, *p* < 0.05) than the control group. Additionally, the malignant group demonstrated significantly elevated HRV parameters, including very low frequency (VLF), low frequency (LF), and high frequency (HF), indicating substantial autonomic dysregulation characterized by parasympathetic predominance. Central triggers (OR = 7.16, 95%CI 1.10–46.73) and convulsive/incontinence manifestations (OR = 19.02, 95%CI 2.81–128.64) were independent risk factors of malignant VVS. **Conclusions:** The age at syncope onset was significantly earlier in children with malignant VVS, and children with malignant VVS exhibited profound autonomic dysregulation characterized by significant parasympathetic predominance. Finally, children with episodes induced by central triggers and accompanied by incontinence or convulsions were at a higher risk of asystole.

## 1. Introduction

Syncope is a common emergency in children and adolescents, the most common form of which is vasovagal syncope (VVS) [1,2]. In most cases, a patient with VVS shows a self-limited progression and quite good prognosis without leaving any sequelae [3]. A different picture is presented by recurrent and severe VVS that is accompanied by prolonged asystole, which is associated with the risk of physical or mental impairment and is also known as malignant VVS. So far, no clear definition and diagnostic criteria for malignant VVS have been established. In some cases, researchers have defined malignant VVS as episodes accompanied by cardiac asystole for more than 3 s [4,5]. Though the nature of the disease is benign, there is no final conclusion on whether the malignant form impairs brain or mental development. Its low prevalence and diagnostic challenges underscore the need for heightened clinical vigilance in identifying this high-risk subgroup. It is particularly critical to identify the manifestation features in these patients and closely follow them up. However, there are only a few relevant studies in children with malignant VVS, and the risk factors of this special group have not been revealed. In this study, we compare the demographics, clinical features, and heart rate variability (HRV) indexes of VVS children with or without asystole and investigate the risk factors associated with malignant VVS in children.

## 2. Materials and Methods

### 2.1. Study Design and Subjects

We retrospectively studied children admitted to the Pediatric Syncope Unit, Peking University First Hospital, and diagnosed with VVS from January 2020 to March 2023. Patients were included in the malignant group if they had (1) a diagnosis of VVS, (2) a record of cardiac asystole for more than 3 s during the syncopal episode confirmed by electrocardiography (ECG), and (3) no other diseases that could lead to syncope. The control group included VVS patients with no asystole lasting more than 2 s during the syncopal attacks and who were admitted to the syncope unit in the same period, and patients were matched with the malignant group based on age and sex by 1:4. The diagnosis of VVS referred to the diagnostic criteria established by the 2018 Chinese Pediatric Cardiology Society guideline for the diagnosis and treatment of syncope in children and adolescents [6]. This study was authorized by the Institutional Ethics Committee of Peking University First Hospital (No. 2024423)

### 2.2. Standing Test and Head-Up Tilt Test

#### 2.2.1. Standing Test

The standing test was conducted in a quiet and dimly lit room. The participants were asked to lie supine on a bed for 10 min and then stand upright on their own for another 10 min. During the test, the patients’ heart rate (HR) and blood pressure (BP) were recorded, and a standard three-lead ECG was performed with a Dash 2000 Multi-lead Physiological Monitor (General Electric, New York, NY, USA) in the supine position and in the first, third, fifth, seventh, and tenth minutes.

#### 2.2.2. Head-Up Tilt Test

The test environment was quiet, warm, and dimly lit. Medications that could influence the autonomic nervous system were avoided for at least 3 days before the test. The patients were asked to fast overnight prior to the HUTT. The subjects were observed for 10–20 min while assuming a supine position on a tilt table (SHUT-100A, Standard, Jiangyin, Jiangsu, China; and ST-711, Juchi, Beijing, China). Then, the tilt table was tilted 60 degrees, and the HR, BP, and ECG were continuously monitored for 45 min or until a positive outcome was noted.

### 2.3. Clinical Data Collection

The clinical characteristics of both groups were collected; this included the patients’ gender, age at admission, height, body weight, body mass index (BMI), basal heart rate, systolic blood pressure (SBP), and diastolic blood pressure (DBP). A detailed medical history was taken; this included the patients’ age at syncope onset, course of disease, number of episodes, predisposing factors, premonitory symptoms, accompanying symptoms, presence of syncope-related injury, and family history of typical VVS episodes. Predisposing factors were classified into two groups—peripheral trigger and central trigger [7]. Peripheral triggers included prolonged standing, postural change, and after exercise. Central triggers included intramuscular injection, venipuncture, emotion, and pain. Examinations of risk factors, such as the 24 h urine volume, 24 h urinary sodium excretion, and flow-mediated vasodilation (FMD), were also conducted. Video electroencephalogram (VEEG) monitoring was additionally performed for malignant VVS patients presenting with syncope episodes accompanied by transient limb convulsions.

The HRV index was obtained using 24 h Holter monitors (Mortara, Milwaukee, WI, USA). Participants were instructed to abstain from vigorous physical activity, technological devices, and emotional arousal. The sample frequency was 10,000 Hz, and the frequency response range was 0.05 to 60 Hz. The HRV was analyzed using an analyzer (H-Scribe 7.0; Mortara, Milwaukee, WI, USA). HRV time domain and frequency domain analysis was performed following calibration, including the standard deviation of all normal-to-normal intervals (SDNN), the standard deviation of the 5 min average normal-to-normal intervals (SDANN), the percentage of differences between adjacent normal-to-normal intervals that are greater than 50 ms (pNN50), the root mean square of successive differences between adjacent normal cycles (rMSSD), very low frequency (VLF), low frequency (LF), high frequency (HF), and the LF/HF ratio. The results of the VLF, LF, HF, SDNN, SDANN, and rMSSD were expressed in milliseconds (ms). The result of pNN50 was expressed in percent (%).

### 2.4. Data Analysis

Statistical analyses were performed with SPSS 26.0 (IBM, Armonk, NY, USA). The Shapiro–Wilk test was used to test the normality of continuous data. For continuous variables following a normal distribution, the data were described as (mean ± SD), and the independent sample *t*-test was used for a comparison between groups. For non-normal distribution continuous variables, the data were described as the median (interquartile range, IQR), and the Mann–Whitney U test was used to test the between-group variance. Categorical data were described as n (%) and analyzed using the χ^2^ test or Fisher’s exact test. *p* < 0.05 was considered statistically significant.

Binary logistic regression analyses were used to identify the risk factors for malignant VVS. Multivariable logistic regression analyses were built, including gender and variables that were significant at the 0.10 level in the univariate analysis. Forward variable selection was utilized to remove nonsignificant variables until all remaining variables were significant at the 0.05 level with two-tailed, unpaired testing. Effect sizes were expressed as the odds ratio (OR) and 95% confidence interval (95%CI).

## 3. Results

### 3.1. Basic Information of Study Patients

A total of 10 patients (four boys, six girls) met the criteria for malignant VVS. The patient demographics and clinical features are depicted in Table 1. Their ages ranged from 8.0 years to 14.0 years (median age of 11.5 years). The mean age at presentation was 7.0 years (range from 3 to 12 years). They all experienced the transient loss of consciousness with acute onset and more than 3 s of cardiac asystole confirmed by an ECG recording during at least one syncopal episode. Another 40 VVS patients (18 boys, 22 girls) without prolonged cardiac asystole and matched by age and gender served as a control group. A history of trauma before syncopal episodes was denied in all cases. There was no history of systemic illness, heart disease, or neurological disease in any of the cases. There were no positive findings during physical examinations.

In the malignant group, eight patients had asystole confirmed by ECG during the standing test or head-up tilt test (HUTT). Two patients developed syncope, with cardiac asystole detected by the Holter ECG monitor during venipuncture. The time of cardiac arrest confirmed by an ECG record ranged from 3.0 s to 11.3 s. Eight patients had convulsions or incontinence during at least one episode.

Comparisons of the patient demographics and clinical features between the malignant group and the control group are depicted in Table 2. There were no significant differences in age at admission, gender, height, body weight, and BMI between the two groups. The age at syncope onset was significantly earlier [7.0 ± 2.7 years vs. 9.7 ± 2.7 years, *p* < 0.05] in the malignant group. Children with malignant VVS had much higher rates of central-triggered episodes (60.0% vs. 17.5%, *p* < 0.05) and higher rates of syncopal episodes accompanied by convulsion or incontinence (80.0% vs. 17.5%, *p* < 0.05). There were no significant differences in the number of episodes, premonitory symptoms, basal heart rate, basal SBP, and DBP between the two groups.

### 3.2. Parameters of Laboratory Tests and HRV Indexes of Study Patients

In the malignant group, the VEEG findings were normal in those who had syncopal episodes accompanied by transient convulsive activity. There were no significant differences in FMD, the 24 h urinary volume, and 24 h urinary sodium excretion between the malignant group and the control group. The two groups varied significantly in several HRV indexes (Table 3). Children with malignant VVS had higher VLF [43.72 (37.25, 56.05) ms vs. 35.50 (32.19, 42.11) ms, *p* < 0.05], higher LF [29.78 (26.21, 40.98) ms vs. 24.17 (21.35, 32.65) ms, *p* < 0.05], and higher HF [29.59 (22.65, 31.75) ms vs. 18.94 (15.40, 23.15) ms, *p* < 0.05]. No statistically significant differences were found between the two groups in SDNN, SDANN, pNN50, and rMSSD.

### 3.3. Risk Factors of Vasovagal Syncope with Asystole

The differences in the parameters that were significant at the 0.10 level between children in the malignant group and control group in the above-mentioned univariate analyses (age at syncope onset, whether induced by central trigger and whether with convulsion or incontinence, rMMSD, VLF, LF, HF, and LF/HF), as well as gender, were assessed using the multivariable logistic regression analyses. Considering that these parameters may correlate with each other, collinearity analysis was performed to screen indicators. The final candidates included gender, age at syncope onset, whether syncope was induced by a central trigger, convulsions or incontinence during syncopal episodes, and an HF value of more than 30 ms.

Table 4 shows the predictors of malignant VVS determined using multivariate regression analysis. Convulsions or incontinence during syncopal episodes and induction by a central trigger were independent predictors of malignant VVS, with odds ratios (95% confidence intervals) of 19.02 (2.81–128.64) (*p* < 0.05) and 7.16 (1.10–46.73) (*p* < 0.05), respectively.

## 4. Discussion

In this study, we explored the features of malignant VVS and tried to identify predictors or risk factors. Our study indicated that there were special clinical features that discriminated malignant VVS from non-malignant VVS. We found that the age at syncope onset was significantly earlier in children with malignant VVS. Children with malignant VVS were more likely to suffer from syncope episodes induced by a central trigger and accompanied by convulsion or incontinence. Moreover, this group exhibited significantly elevated VLF, LF, and HF values, which reflect a disturbance of the cardiac vagal–sympathetic balance characterized by significant parasympathetic predominance. Furthermore, it is worth noting that syncopal episodes with incontinence or convulsions and central triggers were independent risk factors for the malignant form of VVS in children.

In our study, the average age of children with malignant VVS at their first syncope episode was 7.0 years, with the oldest being 12.0 years and the youngest being 3.0 years. According to the Expert Consensus Statement of the Heart Rhythm Society in 2015, the incidence of VVS begins to increase significantly around the age of 11.0, with the median age of children at their first syncope being approximately 14.0 years [8]. An investigation from Changsha showed that the average age of onset for VVS is 13.9 years [9]. Compared with these studies, the characteristic of early onset was also evident in the malignant group in our study.

The most common trigger of VVS is orthostatic stress, which is a peripheral trigger [10]. Moreover, emotional stress, such as fear, pain, loud noises, and blood phobia, can mediate VVS as well [11,12,13], which could be classified as central triggers. In our study, children with malignant VVS were more likely to suffer from syncope episodes induced by central triggers, which was in accordance with Furukaka T’s results in adult patients [14]. In our series, six patients experienced episodes of syncope with confirmed cardiac asystole during HUTT, two patients experienced this during the standing test, and two patients experienced this during intramuscular injection or venipuncture. Both the HUTT and the standing test are provocation tests for the vasovagal reflex. The provocative effect of the HUTT is stronger due to the elimination of leg muscle contraction to compensate for the redistribution of blood. It is easier to trigger syncope or pre-syncope in sublingual nitroglycerin HUTT (SNHUTT) because of the excessive vasodilation that occurs after the sublingual administration of nitroglycerin. When a child fainted in the upright or oblique position, immediate restoration to the supine position was able to normalize venous return and prevent the further deterioration of hemodynamic changes. All six patients in our series obtained relief with this method.

In this study, we found that children with malignant VVS were more likely to suffer from syncope episodes accompanied by convulsions or incontinence. This finding was consistent with previous studies suggesting that seizure-like activities are associated with more severe hemodynamic parameter changes during HUTT and a higher occurrence of asystole [15,16,17]. VVS with asystole is characterized by significant cardiac inhibition. If the arrest is sustained for a sufficiently long period of time, the resulting cerebral hypoperfusion will lead to a loss of consciousness for approximately 6 to 8 s after the last heartbeat [18]. The electroencephalographic tracing of malignant vasovagal syncope illustrates a “slow–flat-–slow” sequence during the syncopal event, indicating severe transient cerebral hypoperfusion [19]. Cerebral ischemia inhibits telencephalon and other cortical structures, leading to the activation of the subcortical structures, especially brainstem reticular formation, and then convulsion develops [20]. In our study, the VEEG findings in malignant VVS patients with convulsive manifestations during episodes were normal, as the recordings were obtained during interictal periods rather than during active syncopal events. Urinary incontinence rarely occurs in VVS children. Zhang Q et al. revealed that urinary incontinence occurred in approximately 56% of cardiac syncope cases versus 2.6% of noncardiac syncope cases [21]. The presence of incontinence could reflect the degree of ischemia and hypoxia, and serves as an alert that reminds one of the necessity of excluding organic heart disease.

Our study indicated that VVS episodes with incontinence or convulsions and central triggers were independent risk factors for the malignant form of VVS. These identified risk factors facilitate evidence-based stratification to prioritize diagnostic evaluation for high-risk cases. In affected children undergoing HUTT, continuous electrocardiographic monitoring with hemodynamic tracking is critical, in addition to mandatory preparedness for emergency intervention to cope with potential asystolic events during provocation.

The autonomic nervous system plays a vital role in the pathogenesis of vasovagal syncope, but its precise role is still under investigation. At least two patterns of cardiac autonomic modulation are believed to exist in VVS. One is characterized by a progressive increase in cardiac sympathetic tone up to the onset of syncope, which could be partially explained by the Bezold–Jarisch reflex [22,23]. The second is characterized by sympathetic inhibition and concomitant vagal dominance [23]. The main mechanism of this type may be baroreflex dysfunction and the overexpression of the M_2_ muscarinic receptors [24,25,26]. Currently, HRV is a commonly recognized approach for evaluating cardiac autonomic function. Unlike adults, only a few investigations have studied the resting HRV parameters in children with VVS. Some reported that children with VVS exhibit significantly increased SDNN, RMSSD and HF values compared to healthy children [27,28]. In comparison, others reported the opposite conclusion or found no differences [29,30,31]. One possible explanation for these discrepancies may be the different subtypes of VVS that were enrolled in different studies [29,32]. The different age ranges and sex ratios may contribute to the conflicting conclusions.

There are even fewer studies on the variations in HRV parameters in children with malignant VVS. Zygmunt A et al. studied the HRV in 73 VVS children, of which 4% were the cardioinhibitory type, finding that children with cardioinhibitory syncope had significantly lower SDNN in comparison to children with the mixed type (114 ms vs. 164 ms, *p* < 0.05) [29]. Moreover, Sun R et al. found no difference in time domain parameters between children with malignant VVS and non-malignant VVS [33], the same as our study. In addition, we found that children with malignant VVS exhibited significantly elevated VLF, LF, and HF values, and the increase in HF was greater than that of VLF and LF. VLF and LF mainly reflect the modulation of the sympathetic nerve, and HF mainly reflects vagus nerve tone [34,35,36,37]. Therefore, we speculate that both vagus and sympathetic tone may be increased in children with malignant VVS in the basal state, but the increase in vagal activity is more obvious, so the final integrated result indicates the dominance of vagal activity. Characterizing the autonomic imbalance (parasympathetic predominance) provides a pathophysiological basis for mechanism-driven interventions. In patients exhibiting marked vagal hyperactivity, early escalation to neuromodulatory therapies, such as autonomic nerve function exercise, may be warranted, rather than relying solely on conventional treatment.

Our study has several limitations. The sample size was small, and we did not compare the HRV parameters of VVS children with those of normal controls. Moreover, our study is single-center-based. As mentioned above, the results of HRV analysis can be conflicting; thus, we cannot explain why a significant difference was not observed for the time domain parameters. Further larger multicenter studies are needed to validate our findings.

## 5. Conclusions

Our findings demonstrate that pediatric patients with malignant VVS exhibit distinct clinical and autonomic profiles characterized by earlier symptom onset, a predisposition to the presence of central triggers, and higher rates of syncopial episodes accompanied by convulsions or incontinence. Heart rate variability analysis revealed profound parasympathetic predominance in these children.

## Figures and Tables

**Table 1:** Clinical presentations of 10 children with malignant vasovagal syncope.

Case	Gender	Age at Admission, Years	Age at Syncope Onset, Years	Frequency, Times/Year	Clinical Condition of Asystole Episodes	Asystolic Time, Seconds	Predisposing Factors of Other Episodes	Premonitory Symptom	Convulsions	Incontinence	Positive Family History
1	M	10	3	Seven times in 7 years	HUTT	7.5	Intramuscular injection, painful stimulus	Amaurosis fugax, Palpitations, Chest distress	Yes	Yes	Yes
2	F	11	8	Four times in 3 years	Standing test	5.2	Prolonged standing	Dizziness, Amaurosis fugax	Yes	No	No
3	M	12	12	Once in 2 months	Venipuncture	11	None	None	No	No	Yes
4	M	12	6	Five times in 6 years	Standing test	11.3	After exercise, prolonged standing	Dizziness, Amaurosis fugax, Abdominal pain, Nausea	Yes	No	Yes
5	F	9	4	Four times in 5 years	HUTT	3	Postural change, emotion, prolonged standing	Dizziness	Yes	No	No
6	M	8	8	Twice in 1.5 months	Venipuncture	4.2	Pain	Amaurosis fugax	No	Yes	Yes
7	F	12	7	Five times in 5 years	HUTT	8	Prolonged standing	Dizziness, Amaurosis fugax, Chest distress	Yes	Yes	No
8	F	13	5	Six times in 9 years	HUTT	3	Prolonged standing, after exercise	Dizziness, Amaurosis fugax, Abdominal pain	No	No	Yes
9	F	11	7	Two times in 3 years	HUTT	3	Pain, postural change	Dizziness, Abdominal pain, nausea	Yes	No	No
10	F	14	10	Three times in 4 years	HUTT	3.5	Prolonged standing, postural change, pain	Dizziness, Amaurosis fugax	Yes	No	No

Note: A positive family history means that family members used to have typical episodes of vasovagal syncope that were caused by typical triggers such as prolonged standing, postural change, and venipuncture with no convulsions or incontinence and leaving no sequela; M, male; F, female; HUTT, head-up tilt test.

**Table 2:** A comparison of the clinical characteristics of children with malignant VVS and the control group.

	Malignant Group (N = 10)	Control Group (N = 40)	t/Z/χ^2^ Value	*p* Value
Age at admission, years	11.5 [10.0, 12.3]	12.0 [10.0, 13.00]	−0.320	0.749
Gender			0.721	0.490
Male, n (%)	4 (40.0)	18 (45.0)	-	-
Female, n (%)	6 (60.0)	22 (55.0)	-	-
Height, cm	156.5 [145.3, 164.8]	161.5 [147.0, 167.0]	−0.644	0.520
Body weight, kg	47.5 ± 17.6	46.2 ± 10.8	−0.304	0.684
BMI, kg/cm^2^	19.2 [15.0, 30.7]	17.8 [16.2, 21.1]	−0.243	0.808
Age at syncope onset, years	7.0 ± 2.7	9.7 ± 2.7	2.868	0.009
Course of disease, months	54.0 [23.8, 75.0]	9.5 [2.0, 24.0]	−2.464	0.014
Number of episodes	4 [2, 5]	3 [2, 4]	−1.039	0.299
Predisposing factor				
Peripheral trigger, n (%)	7 (70.0)	39 (97.5)		0.022 ^a^
Prolonged standing, n (%)	6 (60)	29 (72.5)	0.149	0.700
Postural change, n (%)	3 (30.0)	21 (52.5)	0.846	0.358
After exercise, n (%)	2 (20.0)	2 (5.0)	-	0.174 ^a^
Central trigger, n (%)	6 (60.0)	7 (17.5)	5.464	0.019
Intramuscular injection or venipuncture, n (%)	3 (30.0)	2 (5.0)	3.125	0.077
Emotion, n (%)	2 (20.0)	3 (7.5)	0.347	0.556
Pain, n (%)	4 (40.0)	2 (5.0)	6.262	0.012
Atypical, n (%)	0 (0)	9 (22.5)	1.431	0.232
Premonitory symptom				
Dizziness, n (%)	7 (70.0)	24 (60.0)	0.048	0.827
Amaurosis fugax, n (%)	7 (70.0)	26 (65.0)	0.000	1.000
Tinnitus, n (%)	0 (0)	3 (7.5)	-	1.000 ^a^
Gastrointestinal symptoms, n (%)	4 (40.0)	7 (17.5)	1.231	0.267
Palpation, n (%)	2 (20.0)	5 (12.5)	0.010	0.919
Chest distress, n (%)	2 (20.0)	11 (27.5)	0.006	0.936
Absent, n (%)	1 (10.0)	5 (12.5)	0.000	1.000
Convulsions or incontinence, n (%)	8 (80.0)	7 (17.5)	12.054	0.001
Syncope-related injury, n (%)	2 (20.0)	6 (15.0)	0.010	0.919
Positive family history, n (%)	5 (50.0)	9 (22.5)	1.792	0.181
Basel HR, bpm	65.5 [63.5, 76.3]	70.5 [64.3, 80.0]	−1.057	0.290
SBP, mmHg	108.2 ± 9.2	110.3 ± 11.8	−0.625	0.535
DBP, mmHg	59.0 [55.5, 68.0]	63.5 [60.0, 67.8]	−0.875	0.382

Atypical triggers included prolonged sitting and squatting; gastrointestinal symptoms included abdominal pain and nausea; BMI, body mass index; DBP, diastolic blood pressure; HR, heart rate; SBP, systolic blood pressure. ^a^: Fisher’s exact test.

**Table 3:** A comparison of the parameters of the laboratory tests and HRV indexes between children in the malignant group and the control group.

	Malignant Group (N = 10)	Control Group (N = 40)	t/Z/χ^2^ Value	*p*-Value
FMD, %	10.1 ± 4.6	10.8 ± 3.2	0.551	0.584
24-h urinary volume, mL	1360.0 ± 705.6	1511.5 ± 622.2	0.671	0.505
24-h urinary sodium excretion, mmol/24 h	114.3 [78.9, 156.7]	119.1 [87.0, 53.9]	−0.364	0.716
HRV indexes				
SDNN, ms	150.4 ± 27.1	147.8 ± 26.7	−0.277	0.783
SDANN, ms	138.6 ± 39.1	129.6 ± 26.0	−0.879	0.384
pNN50, %	24.7 [19.2, 27.7]	17.1 [12.4, 27.0]	−1.164	0.244
rMSSD, ms	55.0 [41.3, 58.0]	41.0 [35.3, 51.8]	−1.833	0.067
VLF, ms	43.7 [37.3, 56.1]	35.5 [32.2, 42.1]	−2.110	0.035
LF, ms	29.8 [26.2, 41.0]	24.2 [21.4, 32.7]	−2.086	0.037
HF, ms	29.6 [22.7, 31.8]	18.9 [15.4, 23.2]	−2.838	0.005
LF/HF	1.1 [1.0, 1.3]	1.3 [1.1, 1.5]	−1.689	0.090

FMD, flow-mediated vasodilation; HF, high frequency; HRV, heart rate variability; LF, low frequency; pNN50, percentage of differences between adjacent normal-to-normal intervals that are greater than 50 ms; rMSSD, root mean square of successive differences between adjacent normal cycles; SDANN, standard deviation of the 5 min average normal-to-normal intervals; SDNN, standard deviation of all normal-to-normal intervals; VLF, very low frequency.

**Table 4:** The risk factors for malignant vasovagal syncope in children.

	B	SE	Wald	*p*	OR (95%CI)
Central trigger	1.969	0.957	4.235	0.040	7.16 (1.10, 46.73)
Syncope with convulsion or incontinence	2.946	0.975	9.125	0.003	19.02 (2.81, 128.64)

## Data Availability

The raw data supporting the conclusions of this article will be made available by the authors on request due to private reason.
